# Turmeric Essential Oil Constituents as Potential Drug Candidates: A Comprehensive Overview of Their Individual Bioactivities

**DOI:** 10.3390/molecules29174210

**Published:** 2024-09-05

**Authors:** Adriana Monserrath Orellana-Paucar

**Affiliations:** 1 Nutrition and Dietetics School, Faculty of Medical Sciences, University of Cuenca, Cuenca 010204, Ecuador; adriana.orellanap@ucuenca.edu.ec; 2 Pharmacology and Nutritional Sciences Interdisciplinary Research Group, Faculty of Medical Sciences, University of Cuenca, Cuenca 010204, Ecuador

**Keywords:** turmeric, *Curcuma longa*, turmeric oil, curcuma oil, bioactivity, toxicity

## Abstract

The therapeutic properties of turmeric essential oil have been extensively documented in both preclinical and clinical studies. Research indicates that its primary active compounds are promising candidates for addressing a wide range of pathologies, exhibiting anticancer, anti-inflammation, antioxidant, cardiovascular, hypoglycemic, dermatological, hepatoprotective, neurological, antiparasitic, antiviral, insecticidal, antifungal, and antivenom activities. While numerous compounds possess similar potential applications, the isolated active constituents of turmeric essential oil stand out due to their unique pharmacological profiles and absence of toxicity. This literature review meticulously compiles and analyzes the bioactivities of these constituents, emphasizing their molecular mechanisms of action, reported pharmacological effects, and potential therapeutic applications. The aim of this review is to provide a comprehensive synthesis of currently available clinical and preclinical findings related to individual turmeric essential oil compounds, while also identifying critical knowledge gaps. By summarizing these findings, this work encourages further research into the isolated compounds from turmeric oil as viable drug candidates, ultimately contributing to the development of innovative therapeutic strategies.

## 1. Introduction

*Curcuma longa* L., commonly known as turmeric, belongs to the Zingiberaceae family and is a native Southeast Asian herb. Its dried rhizome powder has been used for centuries as food, spice, or medicine. Due to its significant biological activities, turmeric is considered an attractive source of drug candidates for preventing or treating various diseases. Most of these pharmacological properties are attributed to the extensively studied curcuminoids [1,2,3,4]. Turmeric rhizomes contain around 2–5% curcuminoids, including curcumin, demethoxycurcumin, and bisdemethoxycurcumin [5].

In addition to curcuminoids, turmeric exhibits a wide variety of chemical constituents with diverse pharmacological activities attributed to its active constituents, including antioxidant, anticancer, anti-inflammatory, cardiovascular, immunomodulatory, hepatoprotection, neuroprotective, antimicrobial, antivenom, and chemo-preventive action, among others [5,6].

Turmeric essential oil, responsible for the spice’s aromatic taste and smell, is isolated from *Curcuma longa* rhizomes and does not contain curcuminoids. Dried turmeric rhizome typically includes 3–6% essential oil [7]. Turmeric rhizome essential oil (TEO) is traditionally obtained by hydro-distillation using the conventional Soxhlet technique.

The chemical composition of TEO exhibits a remarkable consistency on a global scale; however, the concentration of individual compounds varies significantly based on factors such as the geographical origin of the crops, the specific part of the plant utilized, and the methods employed during the drying process. Table 1 illustrates the variation in the content of the essential oil extracted from the rhizome, highlighting differences attributable to geographical location [8,9,10,11].

**Figure 1:**
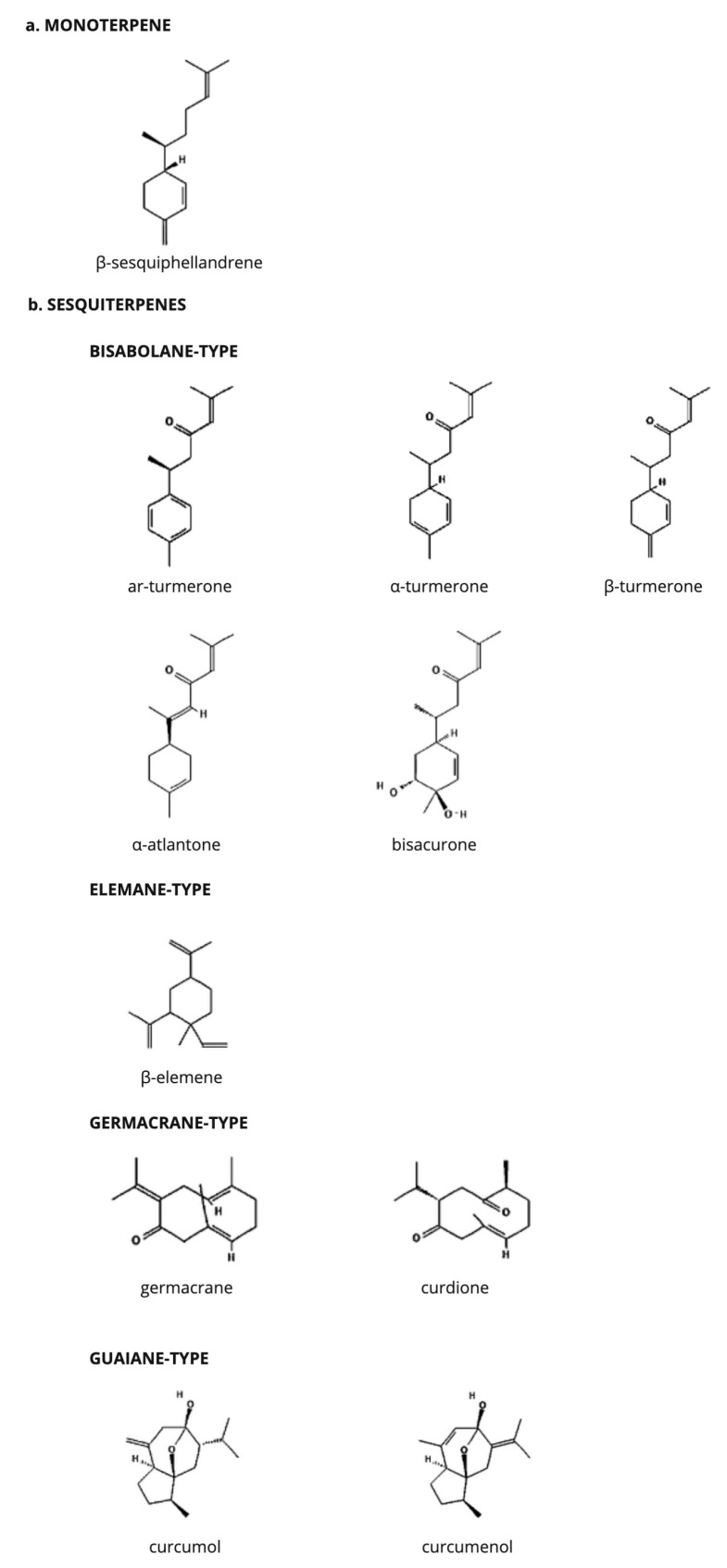
The main chemical constituents of turmeric essential oil with reported bioactive properties.

The primary constituents of TEO with documented pharmacological activities are terpenoids, specifically monoterpenes and sesquiterpenes (Figure 1). Sesquiterpenoids are synthesized by the assembly of three isoprenoid units. Many sesquiterpenoid carbon skeletons originate from the common precursor farnesyl pyrophosphate, undergoing various cyclization processes that are frequently followed by skeletal rearrangements. Consequently, the predominant categories of sesquiterpenes identified in TEO include bisabolane, elemane, germacrane, and guaiane types [12,13]. 

## 2. Bioactivities of Turmeric Essential Oil Constituents

### 2.1. Anticancer Activity

Cancer is a chronic and often fatal disease with a high global mortality rate and generally poor survival outcomes. Cancer-related deaths can result from the heterogeneous nature of cancerous cells or the failure of pharmacological treatments [14]. Interestingly, ar-turmerone, germacrone, and β-elemene have shown potential as anticancer agents. 

Ar-turmerone has been shown to induce apoptosis in human lymphoma and lymphoblast cells through the activation of caspase-3 [15,16]. This bisabolane sesquiterpenoid also downregulates the secretion of growth factor and the phosphorylation of epidermal growth factor [17]. Additionally, ar-turmerone significantly inhibits the TPA-induced upregulation of MMP-9 and COX-2 expression in human breast cancer cells, effectively blocking critical signaling pathways such as NF-kB, PI3K/Akt, and ERK1/2. Importantly, ar-turmerone suppresses TPA-induced invasion, migration, and colony formation in these cells, underscoring its promising therapeutic potential [18]. Furthermore, ar-turmerone induces a highly selective apoptotic process in human leukemia Molt 4B and HL-60 cells [19]. Both α-turmerone and ar-turmerone also enhance the proliferation of peripheral blood mononuclear cells (PBMCs) and stimulate cytokine production. Notably, α-turmerone induces apoptosis in MDA-MB-231 cells and human leukemia cells, as evidenced by a significant reduction in the levels of procaspases-3, -8, and -9 [20].

Regarding benign prostatic hyperplasia, germacrone has been shown to inhibit androgens by selectively targeting the steroid 5-alpha reductase in vitro. This inhibitory effect is attributed to the structural similarity between the α,β-unsaturated carbonyl of germacrone and testosterone. Key factors contributing to its androgenic activity include the conformation of the cyclodecadiene ring and the presence of the α,β-unsaturated ketone/hydroxyl moiety in the germacrone molecule [21]. Additionally, germacrone induces apoptosis in a concentration-dependent manner, with treated cells exhibiting elevated levels of LC3B-II protein and distinctive punctate patterns, indicative of the initiation of protective autophagy. Moreover, germacrone suppresses the phosphorylation of Akt and mTOR in prostate cancer cells [22]. 

On the other hand, β-elemene has demonstrated efficacy in overcoming drug resistance in tumor cells. It inhibits the proliferation of A549/DDP cells in a manner that is dependent on both time and dosage. Furthermore, β-elemene enhances the sensitivity of these cells to cisplatin, effectively reversing drug resistance. Notably, β-elemene induces a reduction in mitochondrial membrane potential, an increase in intracellular reactive oxygen species (ROS) concentration, and a decrease in cytoplasmic glutathione levels. The combined treatment with β-elemene and cisplatin results in elevated protein expression of cytochrome c, caspase-3, and Bad, while concomitantly decreasing the protein levels of Bcl-2 and procaspase-3. This suggests the involvement of a procaspase-3-β-elemene pathway that impacts mitochondrial membrane potential, initiating apoptosis through the release of cytochrome c into the cytoplasm and modulating apoptosis-related genes [23]. 

Collectively, these findings suggest the potential efficacy of turmeric essential oil constituents against cancer cells. However, it is important to note that the primary limitations of these studies stem from their preclinical nature. Therefore, further clinical research is necessary to confirm the positive effects of these isolated compounds on human health. 

### 2.2. Anti-Inflammatory Properties

Inflammation is a complex biological and pathological response that typically arises as a protective mechanism against harmful stimuli, such as infections and tissue injuries, with the aim of maintaining homeostasis within the body. Inflammation can be broadly classified into two categories: acute and chronic. Acute inflammation is a transient and generally beneficial response; however, when inflammation persists over an extended period, it can evolve into chronic inflammation, which is associated with various persistent health conditions, including obesity, diabetes, arthritis, pancreatitis, cardiovascular disorders, neurodegenerative diseases, metabolic disorders, and certain types of cancer [24].

While the anti-inflammatory properties of turmeric have traditionally been attributed to curcumin, bisabolene sesquiterpenes have emerged as a significant class of anti-inflammatory agents [25]. For instance, ar-turmerone has been shown to inhibit CD8+ T cells in the epidermis, leading to the reduced expression of NF-κB and COX-2, as well as the inhibition of p38 MAPK phosphorylation [26]. Additionally, ar-turmerone effectively inhibits critical inflammatory cytokines, including IFN-γ and IL-2, in CD4+ T cells without adversely affecting their proliferation rates upon stimulation [27].

Ar-turmerone also mitigates skin inflammation by lowering the levels of TNF-α and IL-6 while downregulating the mRNA synthesis of IL-17, IL-22, and IL-23. Furthermore, ar-turmerone decreases the production of TNF-α, IL-1β, IL-6, and MCP-1 in Aβ-stimulated microglial cells by inhibiting the NF-κB, JNK, and p38 MAPK signaling pathways [28,29,30]. 

In murine models, germacrone demonstrated anti-inflammatory effects by significantly reducing the expression of pro-inflammatory cytokines IL-6 and TNF-α while promoting the expression of anti-inflammatory mediators such as TGF-β1 and IL-10 [31]. It has been suggested that germacrone plays a crucial neuroprotective role by modulating autophagy through regulation of the PI3K III/Beclin-1/Bcl-2 and PI3K I/Akt/mTOR pathways [32]. 

Curcumol inhibits the LPS-induced nitric oxide (NO) production by suppressing the expression of iNOS mRNA and protein levels, although it does not affect iNOS activity. Furthermore, curcumol reduces the LPS-induced production of TNF-α, IL-1β, and IL-6 at both transcriptional and translational levels, accompanied by a decrease in JNK phosphorylation [33].

The anti-inflammatory potential of these turmeric essential oil constituents has been demonstrated both *in vitro* and in experimental animal models. While historical reports indicate the use of TEO to treat inflammatory diseases [34], further investigation through randomized and controlled clinical studies is warranted to elucidate the underlying mechanisms of action.

### 2.3. Antioxidant Action

Free radicals are generated through the accumulation of reactive oxygen species (ROS) resulting from exposure to oxidizing substances. Free radicals can contribute to the development of various chronic and degenerative diseases. However, the risk of such diseases can be mitigated by employing external antioxidants or enhancing the production of endogenous oxidants [35]. *In vitro* studies have demonstrated the potent antioxidant capacity of ar-turmerone in scavenging free radicals [36]. Additionally, turmerone Q has been shown to inhibit lipopolysaccharide-induced NO production [37]. 

A comparative analysis of the chemical composition and antioxidant activity of essential oils highlighted the significant impact of sample processing, storage, distribution, and preservation on the quality of antioxidant properties. This research compared the antioxidant capabilities of essential oils and crude extracts from the Zingiberaceae family, including turmeric, revealing notable variations in antioxidant activity based on the extraction method employed [38]. Furthermore, the study examined the chemical composition and antioxidant activity of both fresh and dried turmeric samples, finding that the essential oil from fresh turmeric exhibited superior antioxidant activity. Chemical analysis identified α-turmerone as the predominant constituent of this TEO [39].

Antioxidants play a vital role in human physiology and food preservation. Research on turmeric has demonstrated its effectiveness in controlling lipid peroxidation in hamburger and chicken meat during cooking processes [40,41]. Given the antioxidant activity of isolated turmeric compounds, such as ar-turmerone, turmerone Q, and α-turmerone, further characterization of these compounds is essential for potential pharmacological and nutraceutical applications.

### 2.4. Cardiovascular Activity

Endothelial dysfunction and vascular inflammation are key contributors to atherosclerosis, which is the leading cause of cardiovascular disease and a significant risk factor for mortality worldwide [42]. Curdione, a sesquiterpene derived from turmeric essential oil, has demonstrated protective effects against cardiovascular diseases. This compound exhibits potent anticoagulant and anti-thrombotic properties, effectively inhibiting platelet activation. Curdione modulates the expression of vinculin and Talin1 through its interaction with β1-tubulin, thereby influencing the integrin signaling pathway and subsequently restraining platelet activation. Notably, β-1 tubulin serves as a critical target for curdione, suppressing the thrombin-induced activation of human platelets [43]. Furthermore, curdione reduces P-selectin expression in platelet-activating factor (PAF) by elevating cyclic adenosine monophosphate (cAMP) levels and decreasing intracellular calcium mobilization [44].

Similarly, ar-turmerone has been shown to inhibit platelet aggregation triggered by collagen and arachidonic acid, although it does not significantly affect aggregation induced by PAF or thrombin [45]. Additionally, β-elemene has been found to attenuate atherosclerosis and enhance plaque stability through its antioxidative and anti-inflammatory features. In murine models, β-elemene protects against endothelial dysfunction by significantly improving plasma nitrite and nitrate levels, as well as promoting the phosphorylation of endothelial nitric oxide synthase (eNOS) [46]. Moreover, a derivative of β-elemene has demonstrated protective effects on endothelial cells from H_2_O_2_-induced injury by engaging antioxidant mechanisms and activating the PI3K/Akt/eNOS/NO signaling pathways [47].

### 2.5. Hypoglycemic Action

Type 2 diabetes mellitus is the most prevalent chronic metabolic disorder impacting global health. The primary therapeutic approach for managing this condition involves the use of antidiabetic medications aimed at controlling glucose levels. However, the chronic administration of these drugs can lead to clinically significant side effects and drug interactions. Consequently, there is a growing interest in alternative drug candidates with hypoglycemic properties [48]. 

Terpenes and terpenoids are recognized for their antidiabetic activities, which inhibit the action of enzymes responsible for insulin resistance, thereby restoring physiological plasma glucose and insulin levels [49]. The antidiabetic effects of curdione and germacrone have been demonstrated in glucose consumption assays using HepG2 Cells [50]. Additionally, studies in murine models revealed the hypoglycemic potential of ar-turmerone through the activation of peroxisome proliferator-activated receptor gamma (PPAR-g), suggesting a synergistic effect between curcuminoids and sesquiterpenoids such as ar-turmerone [51].

Collectively, these findings indicate that curdione, germacrone, and ar-turmerone offer protective effects against chronic conditions such as insulin resistance and diabetes. However, most research to date has been conducted using cell and animal models, necessitating further clinical trials to establish their therapeutic efficacy. Future studies should focus on prolonged intervention periods and specific endpoints for evaluating health outcomes to comprehensively assess the long-term safety and efficacy of these turmeric essential oil compounds.

### 2.6. Dermatological Application

Turmeric essential oil is widely utilized in cosmetic and pharmaceutical applications due to its antimicrobial, anti-inflammatory, antioxidant, and insect-repelling properties. The major TEO component, ar-turmerone, has alleviated skin inflammation in both *in vitro* and *in vivo* psoriasis models [26,52]. Given that psoriasis is an immune-mediated inflammatory skin disorder, ar-turmerone appears to exert its effects through a dose-dependent suppression of cell proliferation, promotion of apoptosis, and reduction in interleukin (IL)-1β, IL-6, and IL-8 induced by TNF-α in HaCaT cells, as evidenced by the decreased expression levels of Shh, Gli1, and SMO [52]. Additionally, ar-turmerone inhibits CD8+ T cell migration into the epidermis and lowers the expression of NF-κB and COX-2, along with the phosphorylation of p38 MAPK. In imiquimod-induced murine models, the topical application of ar-turmerone reduced the levels of TNF-α and IL-6 while downregulating the mRNA synthesis of IL-17, IL-22, and IL-23 [26]. 

Moreover, ar-turmerone has the potential to serve as a therapeutic agent for hyperpigmentation disorders by inhibiting the expression of tyrosinase and by inactivating α-MSH- and IBMX-induced melanin synthesis and tyrosinase activity [53]. 

Germacrone-type sesquiterpenes have been shown to regulate the UVB-induced mRNA upregulation and protein expression levels of MMP-1, MMP-2, and MMP-3 in human keratinocytes, indicating their potential as photoprotective and anti-aging agents [54]. Furthermore, germacrone may address skin conditions such as acne, hirsutism, and androgenic alopecia due to its inhibitory action on steroid 5-alpha reductase *in vitro* [21]. These findings underscore the promising dermatological applications of ar-turmerone and germacrone as natural bioactive compounds.

### 2.7. Hepatoprotection

Chronic liver diseases can lead to significant injuries, contributing to conditions such as cirrhosis and liver cancer. These chronic injuries stimulate the release of inflammatory cytokines and reactive oxygen species (ROS), while damaged hepatocytes secrete extracellular matrix protein, resulting in fibrosis. The hepatoprotective effect of TEO sesquiterpenes have been demonstrated in a murine model of D-galactosamine-induced liver injury where ar-, α-, and β-turmerone effectively suppressed the elevated levels of lactate dehydrogenase (LDH), alanine aminotransferase (ALT), and aspartate aminotransferase (AST) [55]. 

Ar-turmerone and bisacurone have also shown protective effects against ethanol-induced hepatocyte injury, a common cause of alcohol-related liver damage [56]. *In vitro* studies have indicated that turmeric essential oil sesquiterpenes, including ar-turmerone, β-sesquiphellandrene, and curcumenol, exhibited cytotoxic activity through the inhibition of cell growth and the induction of apoptosis in the HepG2 cell line [57]. 

Curcumol has been shown to effectively inhibit hepatic stellate cells (HSCs), reducing the secretion and expression of POSTN, and inhibiting the NF-kB signaling pathway along with the production of pro-inflammatory factors [58].

Collectively, ar-turmerone, α-turmerone, β-turmerone bisacurone, β-sesquiphellandrene, curcumenol, and curcumol appear to exert hepatoprotective effects by modulating various signaling pathways. Further investigations are warranted to elucidate the molecular mechanisms underlying their protective actions against hepatic pathologies.

### 2.8. Neurological Action

The neuroprotective properties of turmeric essential oil and its constituents are closely associated with their anti-inflammatory and antioxidant activities at the neuronal level [59]. β-elemene has been shown to reduce the expression of pro-inflammatory cytokines, such as tumor necrosis factor-a (TNF-α), interleukin-1β (IL-1β), and IL-6, while mitigating the translocation of nuclear factor-kB (NF-κB) p65 from the cytoplasm to the nucleus in BV-2 cells exposed to lipopolysaccharide. Additionally, β-elemene inhibits the activation of RAC1, mixed-lineage protein kinase 3 (MLK3), and p38 mitogen-activated protein kinase (MAPK), while increasing the phosphorylation of the RAC1 Ser71 site [60]. Germacrone has also improved motor dysfunction, spatial learning issues, and memory deficits induced by traumatic brain injury in murine models, with this mechanism of action involving Nrf2 upregulation and downregulation of the pro-inflammatory protein p-p65 [61]. 

Ar-turmerone and its analogs have demonstrated the ability to inhibit dopaminergic neurodegeneration by activating nuclear factor erythroid 2-related factor 2 (Nrf2) in dopaminergic neurons. Furthermore, ar-turmerone inhibits acetylcholinesterase activity and mitigates dopaminergic neurodegeneration through significant anti-inflammatory action in microglial BV2 cells [62,63]. Given that Parkinson’s disease (PD) is characterized by the loss of dopaminergic neurons in the substantia nigra due to the inflammatory activation of microglia, ar-turmerone is a compelling candidate for the prevention and treatment of PD. Additionally, elevated levels of monoamine oxidase A (MAO-A) are linked to major depression [64], and ar-turmerone has been shown to exert antidepressant-like effects by reducing MAO-A levels and alleviating stress in a murine model [65]. 

The neuroprotective effects of ar-turmerone are further supported by its ability to enhance the survival of primary cerebellar granule neuronal cultures by restraining caspase-3 cleavage. Conversely, in cancer cell lines, ar-turmerone promotes apoptosis and inhibits cell proliferation, indicating a degree of target specificity that may correlate with a lower likelihood of adverse effects [66]. Therefore, further development of ar-turmerone as a potential therapeutic agent for neurological disorders is strongly warranted.

Moreover, the regenerative capacity of endogenous neural stem cells is crucial in the context of neurodegenerative diseases. Ar-turmerone has been shown to promote the dose-dependent differentiation and proliferation of neural stem cells *in vitro* and *in vivo* [67]. Similarly, β-elemene has been reported to stimulate neurite outgrowth and axonal regeneration in ventral spinal cord motoneuronal cells and primary cortical neurons by inhibiting the RhoA signaling pathway, effectively preventing the activation of RhoA kinase, and enhancing the expression of GAP43 [68]. Thus, both ar-turmerone and β-elemene exhibit the potential to regenerate neuronal tissue and demonstrate neuroprotective properties, positioning them as promising candidates for the prevention and treatment of neurodegenerative diseases.

Regarding anticonvulsant activity, bisabolene sesquiterpenoids, including ar-, α-, β-turmerone, and α-atlantone, have displayed anticonvulsant properties in zebrafish and murine models [69]. Further evaluation of ar-turmerone revealed its ability to control seizures in the intravenous pentylenetetrazole (PTZ) and 6-Hz murine models, as well as its ability to decrease the expression of c-fos and brain-derived neurotrophic factor (bdnf), two genes associated with seizure activity in zebrafish. Additionally, the neurological safety of ar-turmerone was assessed in mice using the beam walking test, revealing no adverse effects on balance or motor function. Notably, brain concentration analysis confirmed the ability of ar-turmerone to cross the blood–brain barrier and persist in brain tissue for up to 24 h following intraperitoneal administration [70].

Likewise, curcumol has been shown to enhance GABA-induced currents in cultured mouse hippocampal neurons and human embryonic kidney cells in a concentration-dependent manner. In murine models of seizures induced by PTZ and kainate, curcumol increased the latency period for both clonic and tonic seizures, reduced mortality rates, and decreased seizure susceptibility, indicating that curcumol exerts its anticonvulsant effects by enhancing GABAergic inhibition [71]. 

This comprehensive body of evidence underscores the potential of TEO constituents, particularly ar-turmerone, α-turmerone, β-turmerone, α-atlantone, β-elemene, and curcumol, as therapeutic agents for neurological disorders, warranting further investigation into their mechanisms of action and clinical applications.

### 2.9. Antiparasitic Properties

*In vitro* studies have demonstrated that ar-turmerone exhibits activity against *Plasmodium falciparum* 3D7 (chloroquine-sensitive), with its efficacy being contingent upon the specific stage of the parasite’s life cycle. Notably, ar-turmerone has been shown to inhibit the transition from the ring stage to the trophozoite stage during the intraerythrocytic life cycle of the parasite’s development. This compound displays high cytotoxic specificity, suggesting its potential as a promising non-toxic candidate for antimalarial drug development, warranting further research into the molecular mechanisms underlying its antiplasmodial action [72].

Additionally, turmerones have demonstrated a dose-dependent capacity to inhibit the growth of *Leishmania amazonensis* promastigotes [73]. However, comprehensive research is essential to elucidate the mechanisms that govern their antileishmanial effects.

### 2.10. Antiviral Activity

Influenza is a viral respiratory illness associated with seasonal outbreaks and sporadic pandemics, affecting approximately 10% of the global population annually and resulting in nearly half a million deaths [74]. While vaccine efficacy is generally high, it remains suboptimal in elderly populations [75]. Consequently, there is an urgent need for new influenza vaccines and antiviral therapies. The severity of influenza can be exacerbated by the disruption of cytokine regulation induced by the virus. Bisabolene sesquiterpenoids from turmeric oil have been proposed as potential modulators of this dysregulation, as they may inhibit the expression of virus-induced inflammatory cytokines by regulating the NF-κB/MAPK and RIG-1/STAT-1/2 signaling pathways in vitro [76].

Moreover, germacrone has been shown to inhibit the replication of H1N1 and H3N2 influenza A viruses, as well as influenza B virus, in a dose-dependent manner. *In vitro* studies indicate that germacrone reduces viral protein expression, RNA synthesis, and the production of infectious progeny virus. Additionally, this compound inhibits viral attachment during the early stages of the replication cycle. *In vivo*, germacrone has demonstrated protective effects against lethal infection in mice, significantly reducing viral titers in lung tissue. A synergistic effect was observed when germacrone was combined with oseltamivir in both *in vitro* and *in vivo* models [77]. 

Furthermore, germacrone exhibits antiviral activity against pseudorabies virus (PRV), a member of the Herpesviridae family responsible for various acute infections in animals, particularly pigs. Given the significant public health implications, there is an urgent need for innovative therapeutic options to effectively manage the transmission and severity of PRV infections, as current treatments have shown limited efficacy. In this context, germacrone emerges as a promising candidate, demonstrating the ability to inhibit PRV replication *in vitro* in a dose-dependent manner [78].

Consequently, both bisabolane sesquiterpenes and germacrone represent promising avenues for further development as therapeutic agents or adjuncts in the treatment of influenza and pseudorabies virus infections.

### 2.11. Insecticidal Action

Synthetic insecticides are the predominant method for vector control; however, their widespread use has led to the emergence of resistant strains and significant environmental contamination. Ar-turmerone has demonstrated larvicidal properties and the ability to deter biting by *Aedes aegypti* L. and *Anopheles quadrimaculatus* mosquitoes [79]. Additionally, ar-turmerone exhibits notable larvicidal activity against *Culex pipiens pallens*, inducing disruptions in the myofibrils of ventral muscle cells in larvae. This effect is mediated through an increase in detoxifying enzymes, including carboxylesterase (CarE), glutathione-S-transferase (GST), and cytochrome P450 monooxidases (P450) [80].

Given these findings, long-term studies are essential to elucidate the specificity of ar-turmerone’s insecticidal action and to objectively assess its safety for both the environment and human health.

### 2.12. Antifungal Properties

Dermatophytosis, caused by pathogenic keratin-digesting fungi known as dermatophytes, affects both humans and animals [81]. Timely and effective treatment is crucial to prevent substantial cosmetic and health issues. However, the presence of adverse effects and the emergence of drug-resistant strains underscore the necessity for novel therapeutic agents. In this context, ar-turmerone has demonstrated *in vitro* antidermatophytic activity against the genera *Trichophyton*, *Microsporum*, and *Epidermophyton* [82]. Further clinical assessment of the antifungal properties of ar-turmerone will provide valuable insights into its molecular mechanisms of action, safety profile, and overall efficacy.

### 2.13. Antivenom Activity

The antivenom activity of turmeric essential oil appears to be closely linked to the anti-inflammatory properties of ar-turmerone. This compound has been shown to inhibit lymphocyte proliferation and their natural killer activity. In murine models, ar-turmerone neutralized the hemorrhagic effects induced by *Bothrops jararaca* venom and the lethal impact of *Crotalus durissus terrificus* venom. Moreover, numerous immunological studies have demonstrated that ar-turmerone can inhibit lymphocyte proliferation and the natural killer activity of human lymphocytes [83].

Further investigation into ar-turmerone and its pharmacological targets is essential to fully comprehend its potential for antivenom applications. Additionally, the mechanisms by which this compound operates and its safety in humans require additional exploration.

Table 2 summarizes published research studies concerning the anticancer, anti-inflammatory, antioxidant, cardiovascular, hypoglycemic, dermatological, hepatoprotective, neurological, antiparasitic, antiviral, insecticidal, antifungal, and antivenom properties of the constituents derived from turmeric essential oil.

## 3. Safety of the Bioactive Constituents of Turmeric Essential Oil

TEO exhibits a favorable safety profile when consumed in dietary contexts [7], with no documented cases of toxicity associated with its oral intake. The cytotoxic effects observed in laboratory settings are context-specific and dose-dependent, primarily occurring under conditions such as simulated oncological changes. Importantly, these effects do not translate to adverse outcomes in typical dietary consumption. While the potential cytotoxicity of turmeric essential oil components warrants further research, it is essential to recognize that such effects do not reflect the overall safety of the oil as a food additive or dietary supplement.

Currently, there is a notable lack of clinical studies assessing the individual toxicity of the various constituents of TEO. Notably, only one clinical report has documented a single case of a cutaneous allergic reaction potentially linked to the oral consumption of turmeric essential oil, although the specific association was not specifically analyzed [84]. Given that Ayurvedic medicine traditionally endorses the use of turmeric for treating allergies [85], there is an urgent need for clinical studies to establish the therapeutic range of turmeric essential oil and its active constituents for specific routes of administration. Defining these parameters will enhance our understanding of their safety and pharmacokinetics, thereby facilitating their integration into contemporary therapeutic practices.

## 4. Conclusions

This review provides a comprehensive overview of the intricate pharmacological characteristics of the components found in TEO and their potential applications for preventive and therapeutic purposes. Among its chemical constituents, sesquiterpenes represent the predominant group in turmeric essential oil, demonstrating a diverse array of noteworthy bioactivities, including anticancer, anti-inflammatory, antioxidant, cardiovascular, hypoglycemic, dermatological, hepatoprotection, immunological, antiparasitic, antiviral, insecticidal, antifungal, and antivenom properties.

The primary focus of this work was to elucidate the mechanisms that potentially underlie the attributes of monoterpenes, bisabolanes, germacranes, elemanes, and guaianes found in TEO. It is noteworthy that some of these isolated constituents exhibit similar effects or activity pathways, suggesting possible synergistic interactions when co-administered. Furthermore, studies have reported additive effects when isolated compounds are utilized alongside commercially available pharmaceuticals, presenting an intriguing avenue for investigation. Such studies may offer the potential to reduce medication dosages, particularly in the context of chronic conditions, thereby minimizing adverse effects, enhancing therapeutic outcomes, and improving medication adherence.

Most of the studies discussed in this review are preclinical in nature. Therefore, further clinical investigations are imperative to achieve a more comprehensive understanding of the pharmacokinetic profile, therapeutic index, efficacy, and safety of the compounds isolated from turmeric essential oil.

## Figures and Tables

**Table 1:** Variations in major compound content of turmeric essential oil.

	India	Korea	Ecuador
ar-turmerone	16.7–25.7%	19.54–32.24%	1.08–45.5%
α-turmerone	30.1–32.0%	3.72–6.50%	13.4–19.8%
β-turmerone	14.7–18.4%	2.86–5.60%	7.35%
α-zingiberene	1.5–4.2%	-	5.3%

**Table 2:** Summary of research on the bioactive properties of turmeric essential oil constituents.

Bioactivity	Compound	Study Design	Sample/Subject	Dose	Route	Effect	Reference
Anticancer	Ar-turmerone	*In vitro*	U937 cells	61–84%	NA	Apoptosis induction through caspase-3 activation involving Bax and p53 proteins, not Bcl-2 and p21	[15]
						Cell death mediated through activation of mitochondrial cytochrome c and caspase-3	
		*In vivo*	P388D1 lymphoblast cell implanted tumors in mouse model	200–300 mg/kg	i.p.	Immune activity enhancement and inhibition of P388D1 lymphocytic leukemia	[16]
						Increase in T-lymphocyte and B-lymphocyte proliferation activities	
						IL-2 production activity increase	
		*In vitro*	Human breast MDA-MB-231 cells	10–30 mM	NA	Inhibition of MMP-9 and COX-2 via NF-kB	[18]
						Suppression of TPA-induced invasion and migration	
		*In vitro*	Human leukemia Molt 4B and H60 cells	30 µg/mL	NA	Selective apoptosis in human leukemia cells	[19]
	Ar-turmerone and α-turmerone	*In vitro*	Human cancer cell lines: HepG2, MCF-7, MDA-MB-231; human skin fibroblast cell line: Hs-68	11.0–41.8 μg/mL	NA	Inhibition of cancer cell proliferation and apoptosis induction	[20]
						Stimulation of immune cell proliferation and cytokine production	
	Germacrone	*In vitro*	Prostate cancer cell lines: PC-3 and 22RV1	30 to 480 mM	NA	Apoptosis and autophagy induction in prostate cancer cells	[22]
						Inhibition of Akt/mTOR signaling pathway, leading to cell death	
	β-elemene	*In vitro*	A549/DDP lung cancer cells	20 μg/mL	NA	Reversal of lung cancer pharmacoresistance via mitochondrial apoptosis pathway	[23]
						Enhancement of cisplatin sensitivity and apoptosis induction in A549/DDP cells	
Anti-inflammation	Ar-turmerone	*In vitro*	CD4+ T cells	10 mM	NA	Suppression of IFN-g and IL-2 production in T cells.	[27]
						Anti-inflammatory effects without affecting IL-4, IL-5, or T-cell expansion	
		*In vitro*	BV-2 microglial cells	5 μM	NA	Suppression of LPS-induced neuroinflammatory molecules in microglia	[28]
		*In vitro*	BV2 microglial and HT-22 hippocampal cells	5–20 μM	NA	Inhibition of neuroinflammatory molecules and ROS production in microglial cells	[30]
						Protection of hippocampal cells from neuronal toxicity	
						Suppression of NF-κB, JNK, and p38 MAPK signaling pathways	
	Turmerones (including ar-turmerone)	*In vitro/In vivo*	QR-32 cells/mouse	0.2–100 nM/500 ppp	NA/p.o.	Significant tumor growth reduction in mice	[29]
						Inhibition of inflammation-related carcinogenesis in mouse model	
						Maintenance of a reducing environment at inflammatory lesions	
						Suppression of iNOS and 8-OHdG expression	
	Germacrone	*In vivo*	Human type II-like alveolar epithelial cells A549/rats	50–150 μM/10 mg/kg	NA/i.p.	Cell apoptosis reduction and promotion of cell viability	[31]
						Attenuation of LPS-induced pathological changes and pulmonary edema in rats	
						Decrease in IL-6 and TNF-α and increase in TGF-β1 and IL-10	
		*In vitro*	PC12 cells	20–80 μM	NA	Inhibition of autophagy in PC12 cells, improving cell viability	[32]
						Control of PC12 cell injury caused by OGDR	
	Curcumol	*In vitro*	Murine macrophage RAW264.7 cell line	12.5–200 μM	NA	Inhibition of NO production, TNF-α, IL-1β, and IL-6	[33]
						Suppression of JNK-mediated AP-1 pathway, targeting inflammation mediators	
Antioxidant	Turmerone Q	*In vitro*	RAW264.7 cell line	Not provided	NA	Inhibition of NO production in macrophages	[37]
Cardiovascular	Curdione	*In vitro/In vivo*	Human platelets	100 μM	NA	Inhibition of platelet activation by targeting b1-tubulin and vinculin	[43]
						Downregulation of Talin1 and b1-tubulin proteins	
		*In vitro/In vivo*	Human platelets/mouse	20–1000 μM/50–200 mg/kg	NA/p.o.	Inhibition of PAF and thrombin-induced platelet aggregation	[44]
						Increase in cAMP levels and suppression of intracellular Ca^2+^ mobilization in platelets	
	Ar-turmerone	*In vitro*	Rabbit platelets	100 µg/mL	NA	Inhibition of platelet aggregation induced by collagen and arachidonic acid	[45]
						More potent activity than aspirin against collagen-induced platelet aggregation	
	β-elemene	*In vivo*	C57BL/6 mice	Not provided	intragastrical	Enhancement of antioxidative defense and reduced lipid peroxidation in atherosclerosis	[46]
						Increase in plasma nitrite and nitrate levels and eNOS phosphorylation in ApoE^−/−^ mice	
		*In vitro*	Human umbilical vein endothelial cells	0.1, 1, and 10 μmol/L	NA	Antioxidant activity superior to vitamin E	[47]
						Protection against oxidative stress by inhibiting ROS production and signaling pathways	
Hypoglycemic	Ar-turmerone	*In vivo*	Type-2 diabetic KK-Ay mice	0.1–0.5 g/100 g of diet	p.o.	Control of blood glucose increase	[49]
						Stimulation of human adipocyte differentiation and PPAR-γ ligand-binding activity	
Dermatological	Ar-turmerone	*In vivo*	IMQ-induced psoriasis-like BALBc mice	0.4–40 mg/kg/day	topical	Inhibition of CD8 T cells, NF-kB, and proinflammatory cytokines	[26]
						Reduction in TNF-a, IL-6, IL-17, IL-22, and IL-23 levels	
		*In vitro*	HaCaT cells	5–30 μM	NA	Reduction in cell proliferation and inflammatory cytokine expression	[52]
		*In vitro*	B16F10 murine melanoma cells	5–40 μM	NA	Inhibition of a-MSH and IBMX-induced melanogenesis by suppressing CREB	[53]
						Expression reduction in tyrosinase, TRP-1, and TRP-2 in cells	
	Germacrone	*In vitro*	HaCaT cells	5–10 μM	NA	Inhibition of UVB-induced MMP upregulation in keratinocytes	[54]
Hepatoprotection	Ar-, α-, and β-turmerone	*In vivo*	Wistar rats	0.5%	p.o.	Reduced liver injury markers in rats	[55]
						Downregulation of LDH, ALT, and AST increased levels triggered by D-GalN treatment	
	Ar-turmerone and bisacurone	*In vitro*	Hepatocytes isolated from Sprague–Dawley rats	1–6 μM	NA	Preventive effects against ethanol-induced injury in primary cells	[56]
	Ar-turmerone, β-sesquiphellandrene and curcumenol	*In vitro*	Hepatoma cell line (HepG2)	15–2000 μg/mL	NA	Inhibition of hepatoma cell growth	[57]
	Curcumol	*In vitro*	Human hepatic stellate cells (HSCs)	20–45 μM	NA	Inhibition of HSC migration and adhesion by regulating NF-kB	[58]
		*In vivo*	ICR mice	30 mg/kg	p.o.	Reduction in periostin (POSTN) secretion and expression in HSCs	
Neurological	β-elemene	*In vitro*	Microglial cell line BV-2	1–25 μM	NA	Alleviated sepsis-associated encephalopathy by inhibiting RAC1/MLK3/p38 pathway	[60]
		*In vivo*	C57BL6 mice	10–40 mg/kg	i.p.	Reduced p38 MAPK phosphorylation and pro-inflammatory cytokines in hippocampus	
						Improved learning and memory in septic mice	
		*In vivo*	Sprague–Dawley rats	80–320 μg/kg	Not specified	Enhancement of neurite outgrowth and GAP-43 expression	[68]
						Inhibition of RhoA kinase activation, promoting locomotor recovery	
						Lesion cavity area reduction and sparing of white matter	
						Significant upregulation of GAP-43 expression	
	Germacrone	*In vivo*	C57BL6 mice	5–20 mg/kg	i.p.	Enhanced motor function and memory, reduced neuroinflammation and oxidative stress	[61]
						Reduced neuronal apoptosis and microglial activation in a dose-dependent manner	
						Increased Nrf2 expression and inhibition of p-p65 expression	
	Ar-turmerone	*In vitro*	Murine microglial BV2 cells	20 μM	NA	Protection of dopaminergic neurons through Nrf2 activation	[62]
						Inhibition of microglial activation and neurodegeneration prevention	
		*In vitro*	Human breast MDA-MB-231 cells	50–250 μM	NA	Acetylcholinesterase inhibition	[63]
		*In vivo*	ICR mice	1.25–5.0 mg/kg	p.o.	Reduced immobility time in mouse forced swimming test and tail suspension test	[65]
						Increased levels of monoamines in various brain regions	
						Decreased MAO-A activity in the frontal cortex and hippocampus	
		*In vitro*	Neural stem cells	1.56–25 μg/mL	NA	Induction of neural stem cell proliferation	[67]
		*In vivo*	Wistar rats	3 mg	intracerebroventricular	Enhanced neuronal differentiation of neural stem cells	
						Mobilization of proliferating neural stem cells from SVZ and hippocampus	
						Promotion of endogenous neural stem cell mobilization in the rat brain	
		*In vitro*	Zebrafish	46 μM	p.o.	Anticonvulsant properties in acute seizure models in mice	[69]
		*In vivo*	C57BI6 and NMRI mice	0.01–50 mg/kg	i.p.	No motor function or balance effects observed in mice post-treatment	
						Rapid absorption and long permanence of ar-turmerone in mouse brains after administration	
	Ar-, α-, β-turmerone, and α-atlantone	*In vivo*	Zebrafish	11–46 μM	p.o.	Electrographic evaluation demonstrated anticonvulsant effects in zebrafish	[70]
			C57BI6 mice	50 mg/kg	i.p.	Anticonvulsant activity in zebrafish and mouse models	
	Curcumol	*In vitro*	Human embryonic kidney cells and primary cultures of mouse hippocampal neurons	10–300 μM	NA	Enhancement of GABAergic inhibition in hippocampus, suppressing neuronal excitability	[71]
		*In vivo*	C57BL6J mice	100 mg/kg	i.p.	Stimulation of GABA A receptors, reducing chemically induced seizure activity in mice	
						Increased GABAergic miniature inhibitory postsynaptic currents in hippocampal slices, affecting amplitude and frequency.	
Antiparasitic	Ar-turmerone	*In vitro*	*Plasmodium falciparum* 3D7	46.8–820.4 µM	NA	Parasite development delayed due to antiplasmodial effect and cytotoxic activity	[72]
	Turmerones	*In vitro*	*Leishmania amazonensis* promastigotes	2.75 µg/mL	p.o.	Significant cellular alterations in *L. amazonensis* promastigotes	[73]
Antiviral	Bisabolane-type sesquiterpenoids	*In vitro*	A549 and MDCK cells	25–100 µg/mL	NA	Inhibition of H1N1 replication in A549 and MDCK cells	[76]
						Regulation of NF-κB/MAPK and RIG-1/STAT-1/2 signaling pathways	
						Reduction in pro-inflammatory cytokine production	
	Germacrone	*In vitro/In vivo*	Madin–Darby canine kidney cells (MDCKs)/BALBc mice	1.6–25 µM/50–100 mg/kg	NA/i.v.	Inhibition of H1N1, H3N2, and influenza B viruses	[77]
		*In vitro*	Vero and PK-1 cells	10–250 µM	NA	Inhibition of PRV replication in a dose-dependent manner	[78]
						Reduction in virus titer and PRV-gB protein level	
Insecticidal	Ar-turmerone	*In vivo*	*Aedes aegypti* mosquitoes	5–25 nmol/cm^2^	p.o.	High biting deterrent activity against mosquitoes	[79]
		*In vivo*	*C. pipiens pallens* larvae	100 p.p.m.	p.o.	Induction of muscle and digestive tissue changes in larvae	[80]
						Larvicidal mechanism involving stomach poison action, unrelated to AChE	
Antifungal	Ar-turmerone	*In vitro*	Dermatophytes	3.90–7.81 µg/mL	NA	Effective antidermatophytic activity	[82]
						Lower MIC values than standard ketoconazole	
Antivenom	Ar-turmerone	*In vivo*	Swiss albino mice	30–70 µg	i.p.	Neutralization of snake venom effects in mice and lymphocytes	[83]
						Inhibition of hemorrhagic activity and lethal effects of snake venoms	
						Blockage of human lymphocyte proliferation and cytotoxicity	

NA (not applicable.); i.p. (intraperitoneal); p.o. (oral); i.v. (intravenous).

## Data Availability

Not applicable.
